# MITF and PU.1 inhibit adipogenesis of ovine primary preadipocytes by restraining C/EBPβ

**DOI:** 10.1186/s11658-016-0032-y

**Published:** 2017-01-17

**Authors:** ChongMei Ruan, Xiu Li, JunJie Hu, Yong Zhang, XingXu Zhao

**Affiliations:** 1 College of Veterinary Medicine, Gansu Agriculture University, Lanzhou, 730070 China; 2 College of Animal Science and Technology, Anhui Agriculture University, Hefei, 230036 China

**Keywords:** Adipogenesis, Lineage-specific transcription factor, Microphthalmia-associated transcription factor, PU box-binding protein, CCAAT-enhancer-binding protein-β

## Abstract

**Background:**

PU box-binding protein (PU.1) is a master gene of hematopoietic lineage and an important specific transcription factor in osteoclast lineage. There is proof of its expression in adipose tissue, and it is known to significantly and negatively affect adipogenesis. However, it is unclear whether there are any other molecules involved in this process.

**Methods:**

We wished to explore the effect of PU.1’s co-activator microphthalmia-associated transcription factor (MITF) on the adipogenic differentiation of ovine primary preadipocytes. The expression vectors pcDNA-MITF and pcDNA-PU.1, and MITF siRNA and PU.1 siRNA were transfected or co-transfected into ovine tail primary preadipocytes. Real-time PCR and western blot analysis were applied to investigate the expression levels of PU.1 and MITF. The morphologic changes in the cells were observed under a microscope at a magnification of × 200 after staining with Oil Red O. The triglyceride (TG) content in cells was also determined after transfection.

**Results:**

MITF and its co-activator PU.1 synergistically exhibited an opposite expression pattern to that of CCAAT-enhancer-binding protein-β (C/EBPβ) during adipogenic differentiation of ovine primary preadipocytes. Before induction of differentiation, overexpression of MITF or PU.1 inhibited the expression of C/EBPβ and adipogenesis in the cells; and knockdown of MITF or PU.1 promoted the expression of C/EBPβ and adipogenesis in the cells. The inhibitory or promotive effect was enhanced when MITF and PU.1 were co-overexpressed or co-silenced. However, when MITF and/or PU.1 were overexpressed after day 2 of differentiation, no changes in adipogenesis of the cells were observed.

**Conclusions:**

MITF and its co-activator PU.1 inhibited adipogenesis of ovine primary preadipocytes by restraining C/EBPβ.

**Electronic supplementary material:**

The online version of this article (doi:10.1186/s11658-016-0032-y) contains supplementary material, which is available to authorized users.

## Introduction

Cell fate manipulation and alternation is a rapidly expanding field with clear applications in human disease treatment and animal performance improvement. Direct conversion of cells from one lineage to another is more feasible and efficient than the low efficiency of generating induced pluripotent stem cells (iPSCs) and limited availability of embryonic stem cells (ESCs) [1]. Lineage-specific transcription factors are regulators that play a pivotal role during lineage trans-differentiation. This has been a major focus of efforts to manipulate cell fate for therapeutic or performance-improving purposes [2–4].

Adipocytes store excess energy in the form of triglycerides and secrete multiple adipokines to influence systemic energy homeostasis [5]. Adipogenesis is a process of cell differentiation by which preadipocytes become mature adipocytes [6]. While marked changes in cell morphology and intracellular components can be observed during adipogenesis, lipid droplets are gradually generated until large central lipid droplets form [7].

Peroxisome proliferator-activated receptor (PPARγ) is the prime regulator of the adipose lineage. It is a well-known transducer in the CCAAT-enhancer-binding protein-β/α–PPARγ (C/EBPβ/α–PPARγ) pathway, which is required to initiate adipogenic differentiation [8]. Recent evidence has revealed that adipogenesis controlled by C/EBPβ/α–PPARγ could be strongly inhibited by PU box-binding protein (PU.1), which is a master regulator of the hematopoietic lineages [4]. Proven to be expressed in the adipose tissue of humans and other animals, PU.1 could suppress the C/EBPβ/α–PPARγ pathway and significantly and negatively influence adipogenesis [9]. However, the underlying mechanism of PU.1 restraint of the C/EBPβ/α–PPARγ pathway and potential involvement of any other factors require further investigation.

Osteoclasts share a common origin with preadipocytes, but they are mutually exclusive in the differentiation process [10]. Microphthalmia-associated transcription factor (MITF) is a basic helix-loop-helix leucine zipper transcription factor involved in lineage-specific pathway regulation of many types of cells, including melanocytes, osteoclasts and mast cells [11]. MITF and its co-activator PU.1 regulated a series of genes involved in osteoclast differentiation [12]. Mice with mutations within the N-terminal DNA-binding basic domain of MITF showed a lack of ability to recruit its co-activator PU.1 and exhibited dysfunctions in normal bone formation [12]. There was tight synergy in the transcription of those genes and/or modulation of their downstream genes. For these reasons, MITF and PU.1 are regarded as a target for treatment of osteopathic conditions, such as bone resorption and osteopetrosis [13].

However, to the best of our knowledge, the role of MITF in adipogenic inhibition caused by PU.1 has been overlooked. In our study, the expression patterns of MITF and PU.1 proteins were analyzed during the adipogenesis of ovine tail primary preadipocytes. We found that MITF and PU.1 proteins displayed an opposite expression pattern to that of C/EBPβ. Before induction of differentiation, overexpression of either MITF or PU.1 inhibited the C/EBPβ–PPARγ pathway and adipogenesis of ovine adipocytes. Knockdown of either MITF or PU.1 enhanced adipogenesis in vitro. This means that although they are not adipose lineage-specifying transcription factors, collectively, MITF and PU.1 synergistically suppressed the differentiation of adipocytes.

## Material and methods

### Animal handling

Healthy small, fat-tailed sheep (20 days old) were provided by the experimental farm of Gansu Agriculture University. All the animal experiments were carried out in accordance with the guidelines for animal tests at the College of Veterinary Medicine, Gansu Agriculture University (Lanzhou, China). The sheep were monitored in a stress-free environment where they were given food and water ad libitum in a humidity- and temperature-controlled room in the Experimental Farm of Gansu Agriculture University (Lanzhou, China).

### Reagents

Oil Red O was ordered from Sigma-Aldrich. Triglyceride GPO-POD assay kits were bought from APPLYGEN. Taq DNA polymerase, T4 DNA ligase, Revert Aid First Strand cDNA Synthesis kits, real-time PCR kits, dNTP and Trizol reagent were purchased from Takara Biotechnology. DMEM/F12, collagenase (type I), fetal bovine serum (FBS) and Opti-MEM were purchased from Gibco (Invitrogen). Suohua-Sofast gene transfection reagents were bought from Xiamen Taiyangma Bioengineering. The qPCR primers *MITF*, *PU.1*, *PPARγ*, *C/EBPβ* and *GAPDH* were designed and synthesized by GenScript. Pure Plasmid Mini kits and DNA gel extraction kits were bought from Bioflux. Anti-MITF, −PU.1, −PPARγ, −aP2, −C/EBPβ and β-tubulin antibodies were purchased from Abcam. Other antibodies used in this study were bought from CWBIOTECH.

### Isolation, culture and differentiation of ovine tail primary preadipocytes

About 300 mg of ovine tail primary preadipocytes were isolated and rinsed with diethyl pyrocarbonate water. The tissue masses were cut with scissors into approximately 1 mm^3^ sections under sterile conditions and digested with type I collagenase (DMEM/F12 + 20 g/l BSA + 1 g/l type I collagenase) for about 60 min at 37 °C in a shaking water bath. DMEM/F12 medium containing 10% FBS was added to stop digestion. The solution was then filtered through 200-μm nylon filters to remove undigested tissue and large cell aggregates, and centrifuged at 2000 rpm for 5 min to separate the floating adipocytes from the pellet of stromal–vascular cells. The pellet was washed twice with serum-free medium. After washing, stromal–vascular cells were resuspended in DMEM/F12 medium containing 10% FBS and counted via hemacytometry. Finally, cells were seeded in culture plates at a density of 5 × 10^4^ cells/cm^2^ and cultured at 37 °C in a humidified atmosphere containing 5% CO_2_. The medium was changed every two days.

The day that cells reached 100% confluence (about two days later) was designated as day 0. Then, the cells were subjected to cocktail-induced differentiation: the cells were incubated with induction solution I (DMEM/F12 with 10% FBS and 10 mg/l insulin, 0.5 mM DEX and 0.5 mM IBMX) for 48 h; then transferred into induction solution II (DMEM/F12 with 10% FBS and 10 mg/l insulin) until the cells differentiated into mature adipocytes. During the differentiation process, the medium was changed every 2 days.

### Transfection of vectors and siRNAs

The expression vectors pcDNA-MITF and pcDNA-PU.1, and MITF siRNA and PU.1 siRNA were transfected or co-transfected into the ovine tail primary preadipocytes using X-treme GENE DNA transfection reagents (Roche) according to the manufacturer’s instructions. Transfection before differentiation was performed when the cells reached 80% of confluence. Transfection after differentiation was performed on day 2 of differentiation after induction solution I was changed.

The pcDNA-MITF vector was provided by Professor William R. Sellers (Brigham and Women’s Hospital, Harvard Medical School). The pcDNA-PU.1 vector was provided by Dr. Qiang Tong (Baylor College of Medicine). MITF siRNA and PU.1 siRNA were designed and synthesized by GenScript.

### Real-time PCR analysis

Total RNA was extracted from ovine tail primary preadipocytes with Trizol Reagent on day 0, 2, 4, 6 and 8 with or without pcDNA-MITF transfection. Three micrograms of total RNA from each category and the control were reverse transcribed to obtain cDNA using the RevertAid First Strand cDNA Synthesis Kit and oligo (dT) 18 primer following the manufacturer’s instructions. All of the PCR primers were designed and synthesized by GenScript. Real-time qPCR was carried out in a final volume of 25 μl containing SYBR Premix Ex Taq polymerase (TaKaRa), 0.4 mM of each primer and 200 ng of cDNA template. Each sample was run in triplicate wells. PCR amplification cycles were performed using the Bio-Rad iQ5 Multicolor Real-Time PCR Detection System and TaKaRa SYBR Premix Ex Taq II kit.

The reactions were initially denatured at 95 °C for 30 s followed by 50 cycles of 95 °C for 5 s, 60 °C for 34 s and 72 °C for 20s. The melting curve analysis was performed at 95 °C for 10 s and 60 °C for 1 min, followed by warm up at a rate of 0.5 °C/10 s until 95 °C was reached. The density of SYBR green I and threshold cycle (Ct) value were analyzed using iQ5 Optical System Software 2.1. The change of transcript abundance for all the tested genes was calculated using the 2^-ΔΔCt^ method. All mRNA amounts were normalized to GAPDH control.

### Western blotting analysis

Proteins were extracted from ovine tail primary preadipocytes according to the protocol described by Pang et al. [14]. About 25 μg of protein of each sample were separated via 12% SDS-PAGE and electro-transferred to PVDF membrane (Millipore) for immunoblot analysis. The following primary antibodies were used: anti-MITF (1:200), −PU.1 (1:200), −PPARγ (1:100), −aP2 (1:400), −C/EBPβ (1:300); and anti-β-tubulin (1:500), which was used as the reference. After incubation with the appropriate HRP-conjugate secondary antibody, proteins were detected and analyzed using the ChemiDoc XRS imaging system and analysis software Quantity One (Bio-Rad).

### Oil red O staining

The cells were washed three times with PBS on day 6, fixed with 4% formaldehyde solution for 1 h, and then stained with 0.5% Oil Red O for 30 min. After being washed twice with PBS, the cells were observed under a microscope at a magnification of × 200.

### Triglyceride content determination

Triglyceride (TG) contents in the mature preadipocytes were determined on day 6 of differentiation using a Triglyceride GPO-POD assay kit according to the to the manufacturer’s instructions. The content was calculated as OD values at 510 nm and the ratio of TG μmol/ng total protein in the cells.

### Statistical analyses

All statistical analyses were performed using SPSS 19.0 statistical software (SPSS Science). The data were presented as means ± S.E.M. Comparisons were made using one-way ANOVA. Significance was set at *p* < 0.05.

## Results

### MITF and C/EBPβ expression patterns during the differentiation of ovine primary preadipocytes

**Fig. 1:**
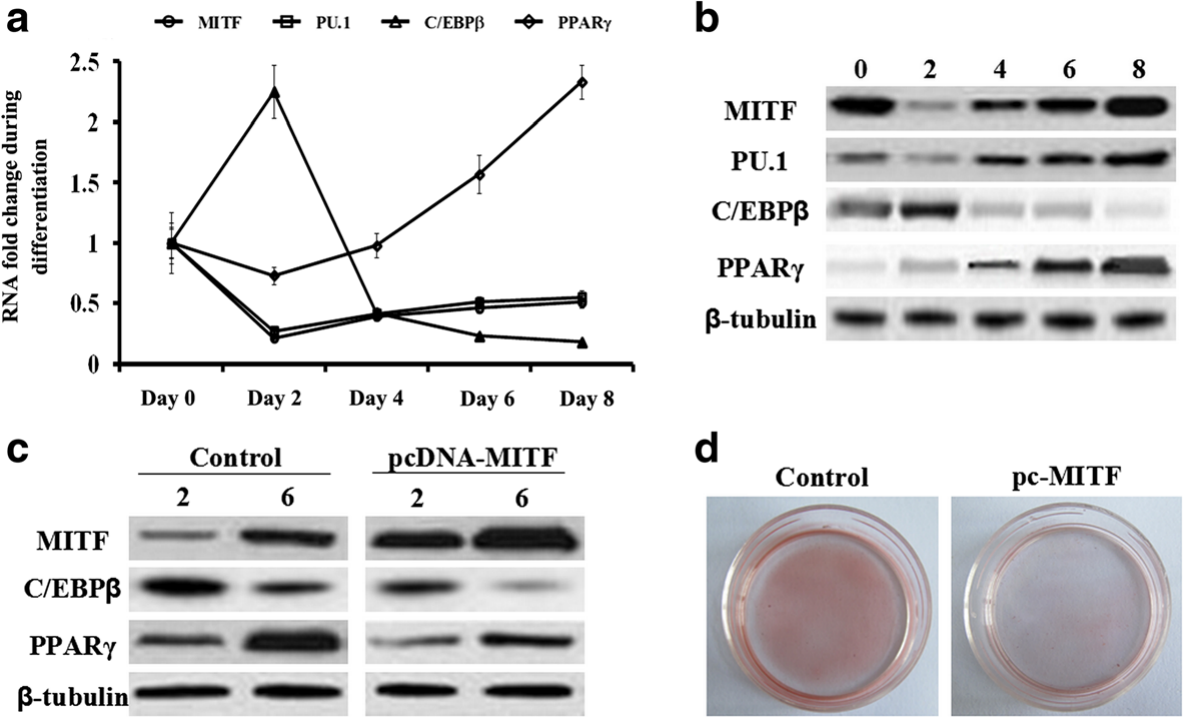
Overexpression of MITF suppressed the expression of C/EBPβ and PPARγ in ovine primary preadipocytes. Primary preadipocytes were isolated from 20-day old small, fat-tailed sheep using the collagenase I digestion method. The cells were seeded in culture plates at a density of 5 × 10^4^ cells/cm^2^ and cultured at 37 °C in a humidified atmosphere containing 5% CO_2_. The cells were then induced to differentiate using the “cocktail” method. On days 0, 2, 4, 6 and 8 during adipogenic differentiation, the cells were collected and their total RNA and proteins were extracted. The expression levels of MITF, PU.1, C/EBPβ and PPAR**γ** were detected via qPCR and western blotting analyses. **a –** The mRNA expression profiles of MITF, PU.1, C/EBPβ and PPAR**γ** during adipogenic differentiation. The mRNA levels of β-tubulin on days 0, 2, 4, 6 and 8 were used as the reference for fold change calculation at each time point. **b –** The protein expression profiles of MITF, PU.1, C/EBPβ and PPAR**γ** during adipogenic differentiation. **c –** Protein expression of C/EBPβ and PPARγ was suppressed by pcDNA**-**MITF transfection. **d –** Oil Red O staining for the ovine primary preadipocytes transfected with pcDNA**-**MITF on day 6 of differentiation

During the adipogenic differentiation of the primary preadipocytes, the expression levels of MITF, PU.1, C/EBPβ and PPARγ were measured using real-time qPCR and western blotting analyses. As expected, the level of PPARγ increased during the differentiation and the expression level of C/EBPβ exhibited a robust increase at the early stage (day 0 to day 2) but then significantly decreased (Fig. 1a and b). The mRNA and protein levels of MITF and PU.1 displayed the opposite pattern to that for C/EBPβ. Their expression sharply decreased at the early stage but elevated from day 2 to the late stage (Fig. 1a and b). These results suggest that MITF and PU.1 might be negatively associated with C/EBPβ during adipogenesis.

### Impact of overexpression of MITF and its co-activator PU.1

MITF was overexpressed in the ovine primary preadipocytes by the *pcDNA-MITF* transfection and the cells were induced to differentiation. On days 2 and 6, the expression levels of C/EBPβ and PPARγ proteins were detected via western blot analysis. The results showed that expression of C/EBPβ and its downstream gene PPARγ were suppressed upon overexpression of MITF (Fig. 1c). Oil Red O staining results on day 6 also indicated that MITF overexpression inhibited adipogenic differentiation (Fig. 1d).

**Fig. 2:**
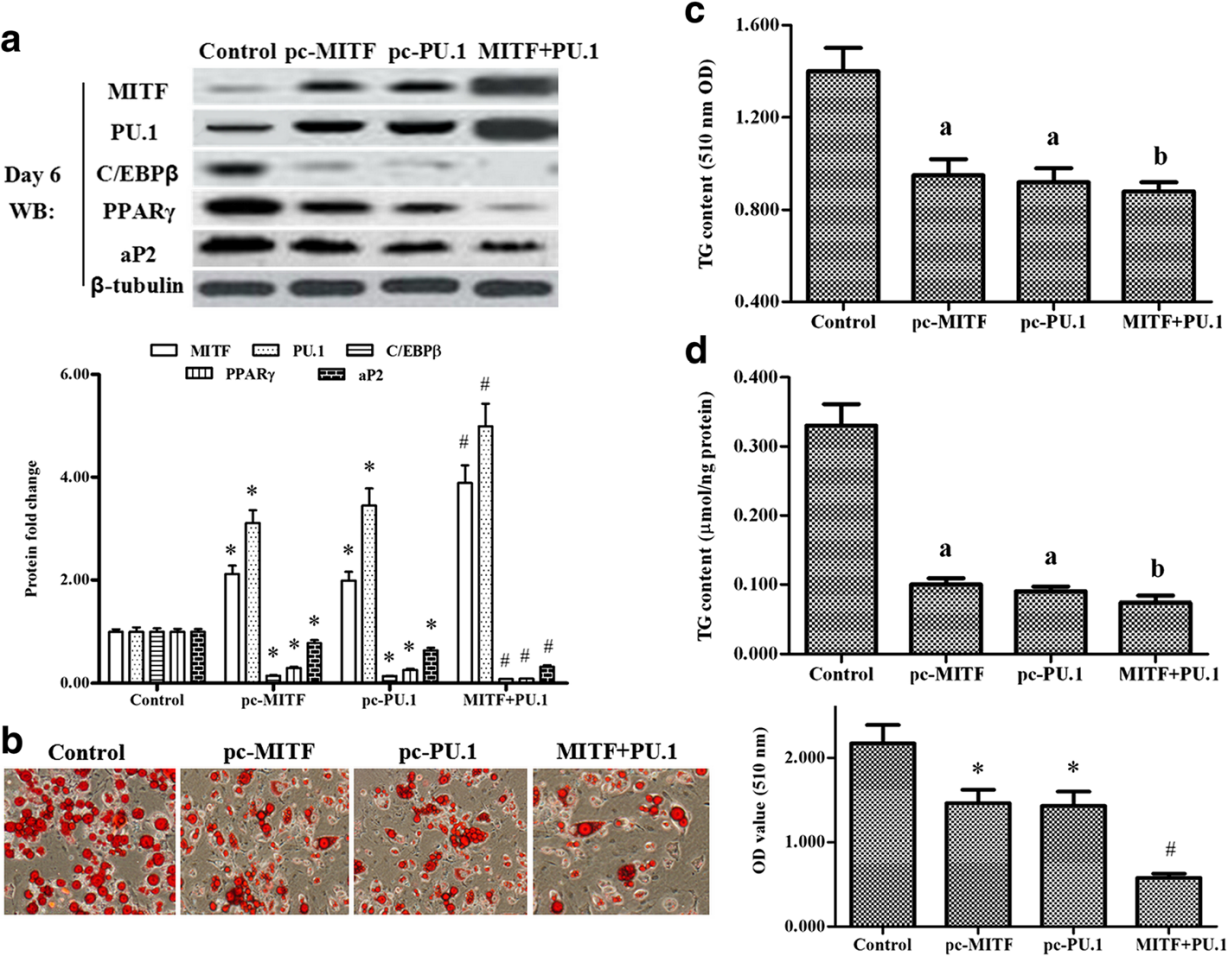
Overexpression of MITF and its co-activator PU.1 inhibited adipogenesis of ovine primary preadipocytes. On reaching 80% confluence, the primary cells were transfected with pcDNA-MITF, pcDNA-PU.1, pcDNA-MITF and pcDNA-PU.1, or nothing. Then the cells were induced to differentiate. On day 6 of adipogenic differentiation, their total proteins were extracted. **a –** Expression of MITF, PU.1, C/EBPβ, PPARγ and aP2 after pcDNA-MITF and/or pcDNA-PU.1 transfection. **b –** Oil Red O staining for the ovine primary preadipocytes transfected with pcDNA**-**MITF and/or pcDNA-PU.1 on day 6 of differentiation. The scale bar is 200 μm. TG content change is shown in (**c**) (510 nm OD value) and (**d**) (μmol/ng protein) after primary preadipocytes were transfected with pcDNA**-**MITF and/or pcDNA-PU.1 on day 6 of differentiation. The same letters on the bar diagram indicated no significant differences between datasets; significant differences indicated by different letters. Significance was set at *p* < 0.05 (*n* = 3), *p < 0.05 vs. the control, ^#^ *p <* 0.05 vs. pc**-**MITF

Next, *pcDNA-MITF* and *pcDNA-PU.1* were transfected alone or co-transfected into the primary preadipocytes prior to induction of differentiation. On days 2 and 6 of differentiation, the expression levels of MITF, PU.1, C/EBPβ and PPARγ were detected using western blot and real-time PCR analyses. The results showed that C/EBPβ and PPARγ were suppressed by MITF or PU.1 overexpression, and the suppression effect was aggravated when MITF and PU.1 were co-overexpressed (Fig. 2a, Additional file 1: Figure S1). MITF or PU.1 overexpression could increase each other’s protein and gene levels. The increase was enhanced when MITF and PU.1 were co-overexpressed (Fig. 2a, Additional file 1: Figure S1). The Oil Red O staining and TG content analyses also revealed that adipogenesis was inhibited by MITF or PU.1 overexpression, and that the inhibition effect was aggravated when MITF and PU.1 were co-overexpressed (Fig. 2b, c, d).

### Impact of knockdown of MITF and its co-activator PU.1

**Fig. 3:**
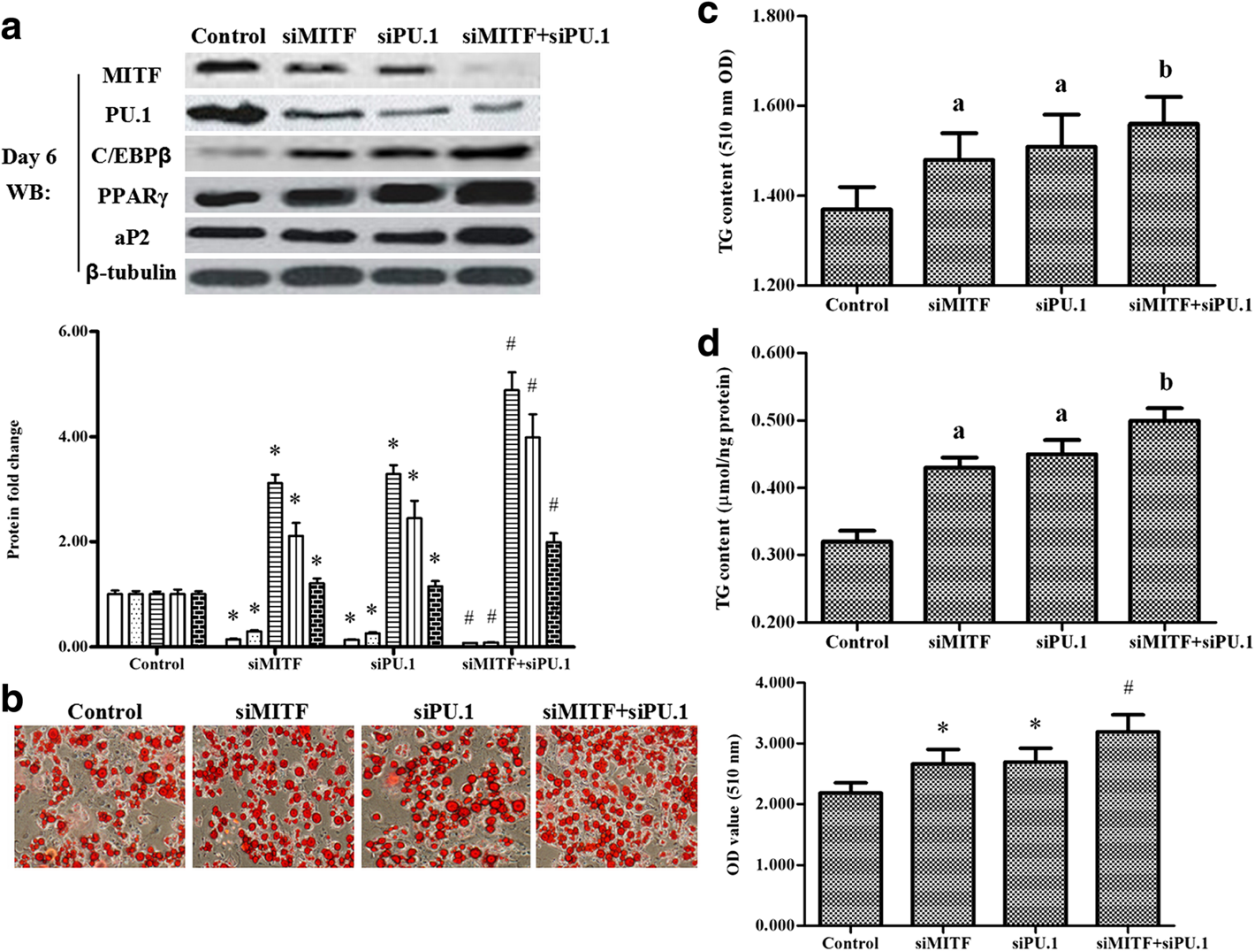
Knockdown of MITF and PU.1 promoted adipogenesis of ovine primary preadipocytes. On reaching 80% confluence, the primary cells were transfected with MITF siRNA, PU.1 siRNA, MITF siRNA and PU.1 siRNA, or nothing. Then the cells were induced to differentiate. On day 6 of adipogenic differentiation, their total proteins were extracted. **a –** Expression of MITF, PU.1, C/EBPβ, PPARγ and aP2 after MITF siRNA and/or PU.1 siRNA transfection. **b –** Oil Red O staining for the ovine primary preadipocytes transfected with MITF siRNA and/or PU.1 siRNA on day 6 of differentiation; The scale bar is 200 μm. The bar diagram (*bottom right*) shows the OD value (510 nm) of the cell matrix containing ovine primary preadipocytes transfected with MITF siRNA and/or PU.1 siRNA on day 6 of differentiation. TG content change is shown in (**c**) (510 nm OD value) and (**d**) (μmol/ng protein) after the primary preadipocytes were transfected with MITF siRNA and/or PU.1 siRNA on day 6 of differentiation. The same letters on the *bar diagram* indicated no significant differences between datasets; significant differences indicated by different letters. Significance was set at *p* < 0.05 (*n* = 3), *p < 0.05 vs. control, ^#^ *p* < 0.05 vs. siMITF

To further investigate the role of MITF in the regulation of adipogenesis, MITF siRNA was transfected alone or co-transfected with the PU.1 siRNA into the primary preadipocytes. Then the cells were induced to differentiation. On days 2 and 6 of differentiation, western blotting and real-time PCR analysis showed that expression of C/EBPβ and PPARγ was enhanced by MITF or PU.1 knockdown, and the enhancement was more distinct when MITF and PU.1 were co-silenced (Fig. 3a, Additional file 2: Figure S2). The knockdown of MITF or PU.1 diminished each other’s protein and gene levels, and the weakening effect was enhanced when MITF and PU.1 were co-silenced (Fig. 3a, Additional file 2: Figure S2). Oil Red O staining and TG content analyses revealed that adipogenesis was promoted by MITF or PU.1 silencing, and the promotion was enhanced when MITF and PU.1 were co-silenced (Fig. 3b, c, d and Additional file 2: Figure S2).

### MITF and PU.1 inhibition of adipogenesis through restraint of C/EBPβ

The above-mentioned results indicate that MITF and PU.1 both inhibited adipogenesis of ovine primary preadipocytes. However, the opposite expression pattern of C/EBPβ and MITF and PU.1 or the effect of MITF and PU.1 overexpression and silencing on adipogenesis and C/EBPβ expression is insufficient evidence to support the conclusion that MITF and PU.1 exerted their function through restraint of C/EBPβ. It is well known that C/EBPβ is just highly expressed and exerts its function at the early stage of adipogenic differentiation. Therefore, if MITF or PU.1 overexpression after day 2 of differentiation could affect the final adipogenic differentiation, it could be concluded that MITF and PU.1 inhibited adipogenesis through other factors but not through restraint of C/EBPβ.

**Fig. 4:**
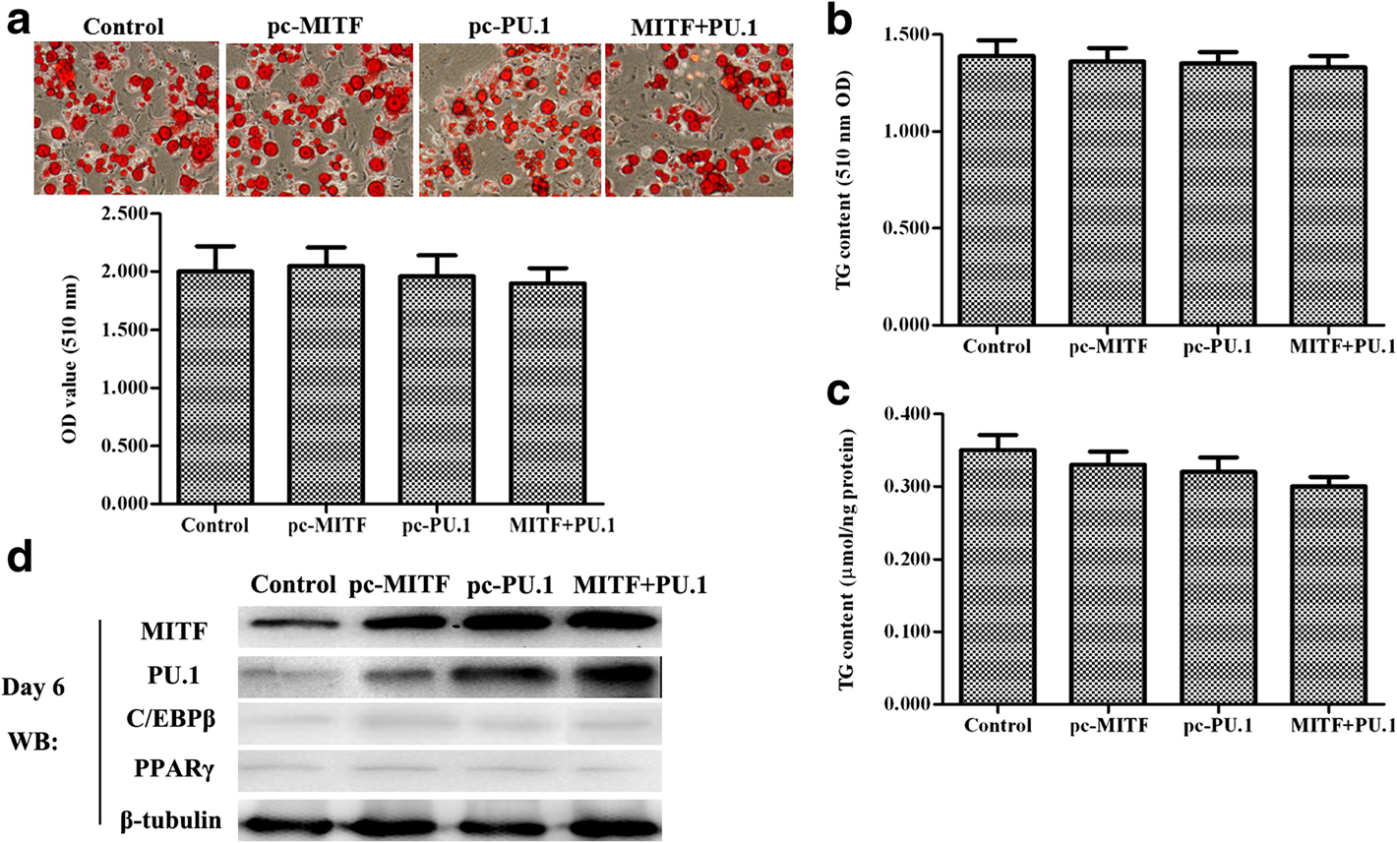
Overexpression of MITF and PU.1 after day 2 of differentiation did not affect the adipogenesis of the ovine primary preadipocytes. The primary preadipocytes were induced to differentiate. On day 2 of differentiation after the induction solution 1 was changed for 4 h, pcDNA-MITF and pcDNA-PU.1 were transfected alone or co-transfected into the cells. Oil Red O staining and TG content analyses were applied to examine the lipid accumulation in the cells on day 6. **a –** Oil Red O staining for the cells on day 6 of differentiation. The scale bar is 200 μm. The bar diagram (*bottom left*) showing the OD value (510 nm) of the cell matrix containing ovine primary preadipocytes transfected with MITF siRNA and/or PU.1 siRNA on day 6 of differentiation. **b –** TG content analysis for cells on day 6 of differentiation (510 nm OD value). **c –** TG content analysis for cells on day 6 of differentiation (umol/ng protein). The same letters on the *bar diagram* indicated no significant differences between datasets; significant differences indicated by different letters. Significance was set at *p* < 0.05 (*n* = 3), *p < 0.05 vs. control, ^#^ *p* < 0.05 vs. siMITF. **d.** Expression of MITF, PU.1, C/EBPβ and PPARγ after pcDNA-MITF and/or pcDNA-PU.1 transfection

Accordingly, ovine primary preadipocytes were induced to differentiate prior to transfection with *pcDNA-MITF* or *pcDNA-PU.1*. On day 2 of differentiation after the induction solution I was changed for 4 h, *pcDNA-MITF* and *pcDNA-PU.1* were transfected alone or co-transfected into the cells. Unlike the previous results, MITF and PU.1 overexpression alone or co-overexpression after day 2 did not alter the adipogenic differentiation of the cells (Fig. 4a, b, c), and no significant expression change of C/EBPβ and PPARγ were observed through western blotting analysis (Fig. 4d), indicating that MITF and PU.1 could inhibit the adipogenesis of ovine primary preadipocytes through restraint of C/EBPβ.

## Discussion

Excessive deposition of adipose in humans or domestic animals is harmful to their health and performance. Controlling the initialization of adipogenic differentiation of preadipocytes, a process known as adipogenesis, is important to avoid excessive adipose deposition. PU.1, which had previously been shown to be expressed exclusively in hematopoietic and lymphoid cells, was also recently detected in the white adipose tissue, where it was shown to play a negative role in adipogenesis [9, 14]. In this study, we found that its co-activator, MITF, a lineage-specific transcription factor in melanocytes, osteoclasts and mast cells, had a restraining effect on C/EBPβ expression and inhibited adipogenesis of ovine primary preadipocytes.

MITF is one of the most well-characterized transcription factors [15]. Numerous studies indicated that through its target genes, it roles in a range of processes, including cell death, DNA replication and repair, membrane trafficking, and mitochondrial metabolism [16]. However, whether MITF had an effect on adipogenesis through its co-activator PU.1 had not been explicitly explored.

Here, we found that MITF was co-expressed with PU.1 in ovine primary adipocytes. The results of our overexpression and knockdown experiments showed that both factors exhibited an opposite expression pattern to that of C/EBPβ during adipogenic differentiation, indicating that MITF co-expressed with PU.1 might inhibit adipogenesis through restraint of the expression of C/EBPβ.

Concurring with most of the findings in reports about the role of PU.1 in adipogenesis [9, 14], our current findings show that overexpression of PU.1 resulted in a notable suppression in the C/EBPβ–PPARγ pathway. However, the underlying mechanism is still not completely understood. MITF and its co-activator PU.1 have recently been designated as a reliable target responsible for osteoclastogenic disorders [13]. It is known that osteoclasts shared a common origin with preadipocytes, but they were mutually exclusive with each other in the differentiation. We speculate that the adipogenic suppression regulated by MITF and PU.1 might be related to their role in osteoclastogenesis. These findings imply that specific transcription factors may have a great impact on the determination of one cell lineage altering into another lineage.

## Conclusion

We found that MITF and PU.1 inhibited adipogenesis of ovine primary preadipocytes through restraint of C/EBPβ. Our findings provided novel insight into the regulation of adipogenesis and cell fate alternation of other lineages through lineage-specific transcription factors.

## Additional files

Additional file 1: Figure S1. Overexpression of MITF and its co-activator PU.1 inhibited the adipogenesis of ovine primary preadipocytes. On reaching 80% confluence, the primary cells were transfected with pcDNA-MITF, pcDNA-PU.1, pcDNA-MITF and pcDNA-PU.1, or nothing. Then the cells were induced to differentiate. On day 2 of adipogenic differentiation, their total proteins and RNA were extracted. A, B – Protein levels of MITF, PU.1, C/EBPβ, PPARγ and aP2 after pcDNA-MITF and/or pcDNA-PU.1 transfection. C, D – mRNA levels of MITF, PU.1, C/EBPβ, PPARγ and aP2 after pcDNA-MITF and/or pcDNA-PU.1 transfection. Significance was set at *p* < 0.05 (*n* = 3), *p < 0.05 vs. control, ^#^ *p <* 0.05 vs. pc**-**MITF. (TIF 462 kb)

Additional file 2: Figure S2. Knockdown of MITF and PU.1 promoted adipogenesis of ovine primary preadipocytes. On reaching 80% confluence, the primary cells were transfected with MITF siRNA, PU.1 siRNA, MITF siRNA and PU.1 siRNA, or nothing. Then the cells were induced to differentiate. On day 2 of adipogenic differentiation, their total proteins and RNA were extracted. A, B – Protein levels of MITF, PU.1, C/EBPβ, PPARγ and aP2 after MITF siRNA and/or PU.1 siRNA transfection. C, D – mRNA levels of MITF, PU.1, C/EBPβ, PPARγ and aP2 after pcDNA-MITF and/or pcDNA-PU.1 transfection. Significance was set at *p* < 0.05 (*n* = 3), *p < 0.05 vs. control, ^#^ *p <* 0.05 vs. pc**-**MITF. (TIF 446 kb)

## Abbreviations

C/EBPβ: CCAAT-enhancer-binding protein-β
C/EBPβ/α: CCAAT-enhancer-binding protein-β/α
ESCs: Embryonic stem cells
FBS: Fetal bovine serum
iPSCs: Induced pluripotent stem cells
MITF: Microphthalmia-associated transcription factor
PPARγ: Peroxisome proliferator-activated receptor
PU.1: PU box-binding protein
