# Supplementary figures and images for: MITF and PU.1 inhibit adipogenesis of ovine primary preadipocytes by restraining C/EBPβ
Source: Cell Mol Biol Lett. 2017 Jan 17;22:2. doi: 10.1186/s11658-016-0032-y (PMC5415744; doi:10.1186/s11658-016-0032-y)

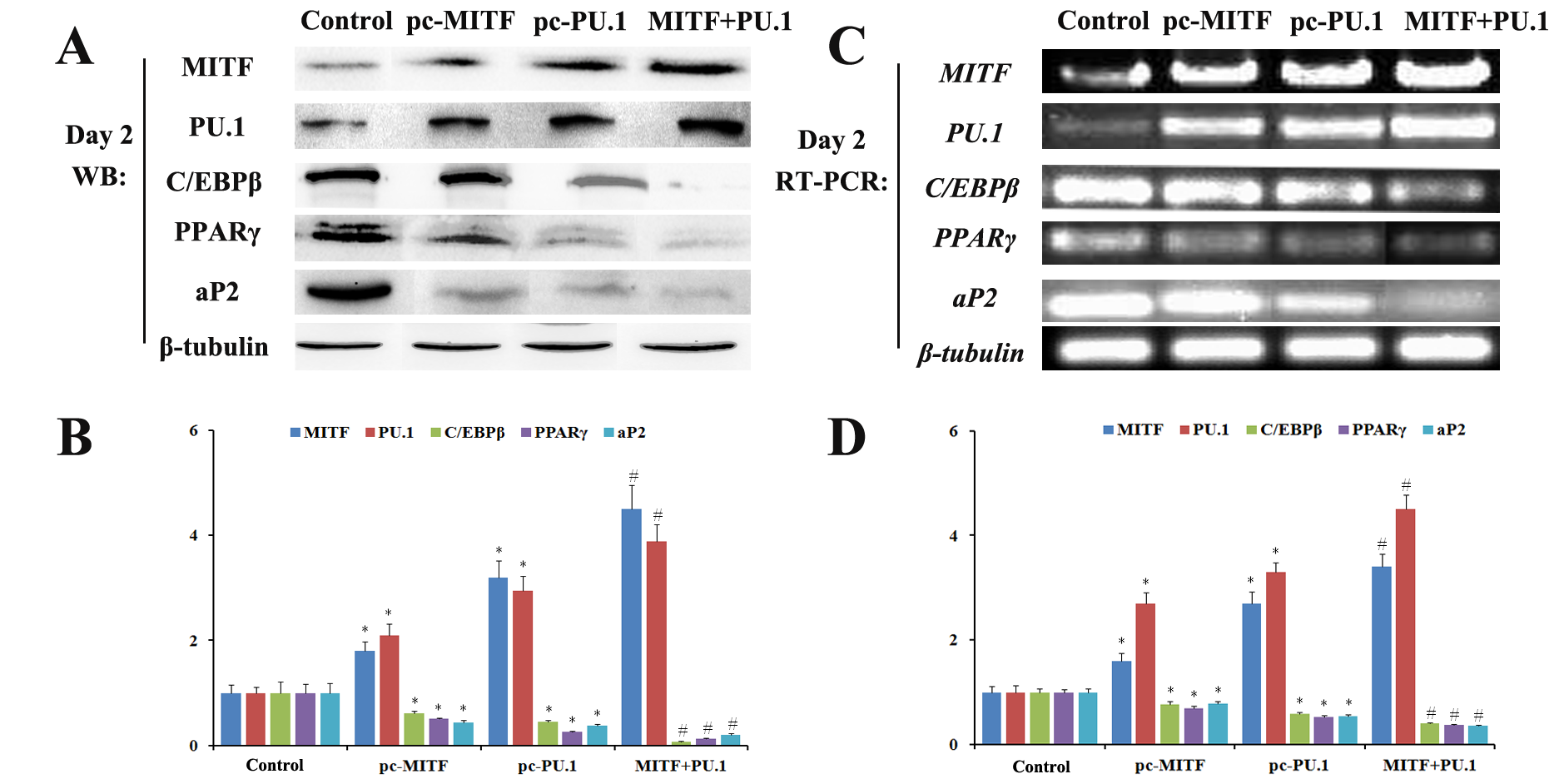

Supplement: Supplementary file 1 — Overexpression of MITF and its co-activator PU.1 inhibited the adipogenesis of ovine primary preadipocytes. On reaching 80% confluence, the primary cells were transfected with pcDNA-MITF, pcDNA-PU.1, pcDNA-MITF and pcDNA-PU.1, or nothing. Then the cells were induced to differentiate. On day 2 of adipogenic differentiation, their total proteins and RNA were extracted. A, B – Protein levels of MITF, PU.1, C/EBPβ, PPARγ and aP2 after pcDNA-MITF and/or pcDNA-PU.1 transfection. C, D – mRNA levels of MITF, PU.1, C/EBPβ, PPARγ and aP2 after pcDNA-MITF and/or pcDNA-PU.1 transfection. Significance was set at p < 0.05 (n = 3), *p < 0.05 vs. control, # p < 0.05 vs. pc-MITF. (TIF 462 kb) [file 11658_2016_32_MOESM1_ESM.tif]

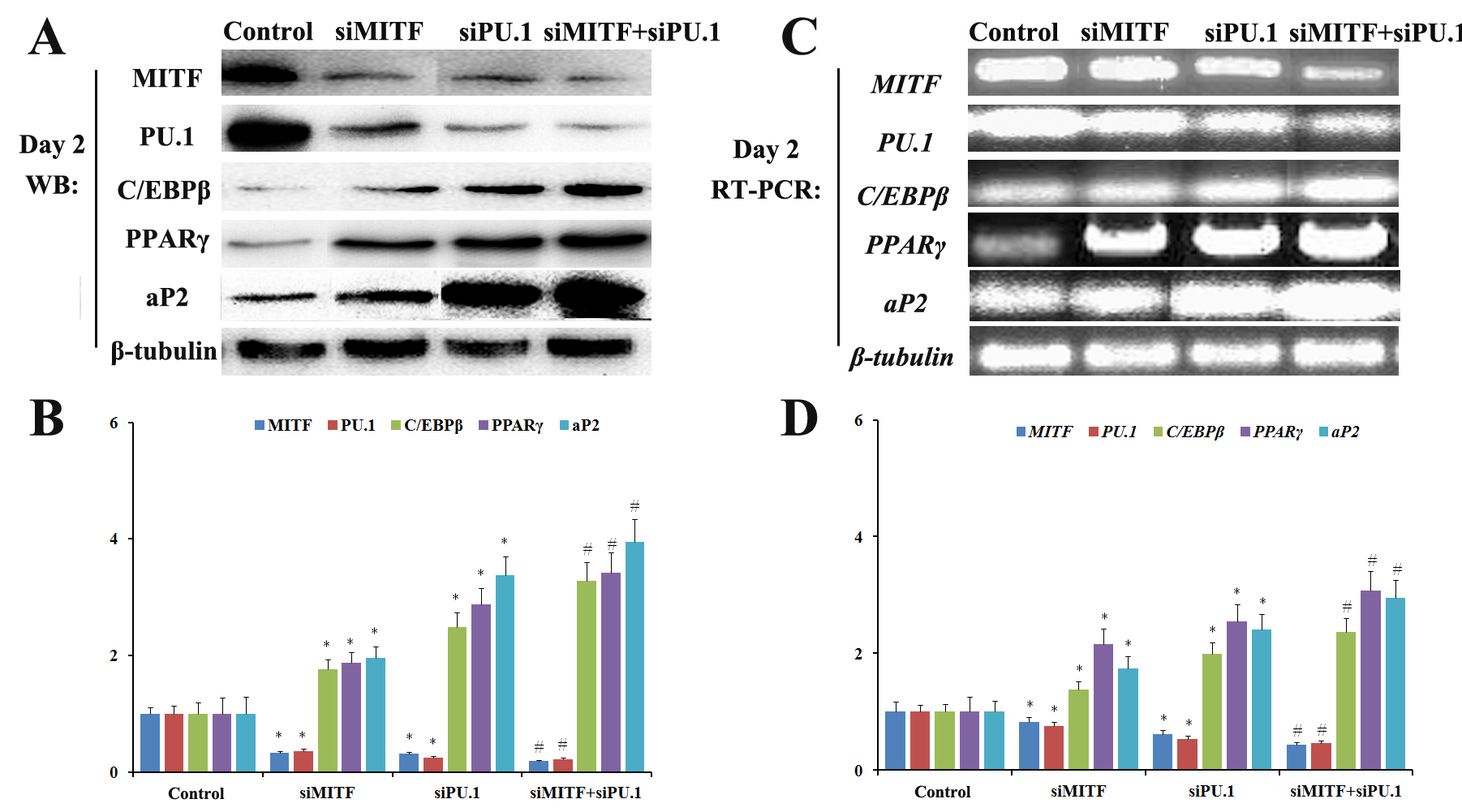

Supplement: Supplementary file 2 — Knockdown of MITF and PU.1 promoted adipogenesis of ovine primary preadipocytes. On reaching 80% confluence, the primary cells were transfected with MITF siRNA, PU.1 siRNA, MITF siRNA and PU.1 siRNA, or nothing. Then the cells were induced to differentiate. On day 2 of adipogenic differentiation, their total proteins and RNA were extracted. A, B – Protein levels of MITF, PU.1, C/EBPβ, PPARγ and aP2 after MITF siRNA and/or PU.1 siRNA transfection. C, D – mRNA levels of MITF, PU.1, C/EBPβ, PPARγ and aP2 after pcDNA-MITF and/or pcDNA-PU.1 transfection. Significance was set at p < 0.05 (n = 3), *p < 0.05 vs. control, # p < 0.05 vs. pc-MITF. (TIF 446 kb) [file 11658_2016_32_MOESM2_ESM.tif]
